# Semiology and determinants of apathy across neurodegenerative motor disorders: A comparison between amyotrophic lateral sclerosis, Parkinson’s and Huntington’s disease

**DOI:** 10.3389/fnagi.2022.1031908

**Published:** 2022-11-02

**Authors:** Barbara Poletti, Federica Solca, Sabrina Maffi, Silvia Torre, Laura Carelli, Edoardo Nicolò Aiello, Roberta Ferrucci, Alberto Priori, Alessia Monti, Federico Verde, Nicola Ticozzi, Simone Migliore, Eugenia Scaricamazza, Melissa Casella, Ferdinando Squitieri, Andrea Ciammola, Vincenzo Silani

**Affiliations:** ^1^Department of Neurology and Laboratory of Neuroscience, IRCCS Istituto Auxologico Italiano, Milan, Italy; ^2^Huntington and Rare Diseases Unit, CSS-Mendel Institute, Fondazione IRCCS Casa Sollievo della Sofferenza Research Hospital, San Giovanni Rotondo, Italy; ^3^Ph.D Program in Neuroscience, School of Medicine and Surgery, University of Milano-Bicocca, Monza, Italy; ^4^Department of Health Sciences, Aldo Ravelli Center for Neurotechnology and Experimental Brain Therapeutics, International Medical School, University of Milan, Milan, Italy; ^5^ASST Santi Paolo e Carlo, San Paolo University Hospital, Milan, Italy; ^6^IRCCS Ca’ Granda Foundation Maggiore Policlinico Hospital, Milan, Italy; ^7^Department of Neurorehabilitation Sciences, Casa di Cura del Policlinico, Milan, Italy; ^8^Department of Pathophysiology and Transplantation, “Dino Ferrari Center,” Università degli Studi di Milano, Milan, Italy; ^9^Italian League for Research on Huntington Foundation, Rome, Italy

**Keywords:** apathy, amyotrophic lateral sclerosis, Parkinson’s disease, Huntington’s disease, neuropsychology

## Abstract

**Background:**

The semiology and determinants of apathy are largely unknown across amyotrophic lateral sclerosis (ALS), Parkinson’s disease (PD), and Huntington’s disease (HD), due to both motor and non-motor confounders. This study thus aimed at (1) profiling apathy in ALS, PD, and HD and (2) exploring its clinical determinants.

**Materials:**

Consecutive ALS (*N* = 99), PD (*N* = 73), and HD (*N* = 25) patients underwent a motor-free assessment of apathy (Dimensional Apathy Scale, DAS), global cognition, anxiety and depression. Function was assessed through disease-specific scales. The DAS was also completed by *N* = 101 healthy controls (HCs). Between-group comparisons on DAS scores were implemented by covarying for all applicable confounders. Predictive models on DAS scores were built through multiple, stepwise regressions.

**Results:**

Parkinson’s disease and HD, but not ALS, patients were more apathetic than HCs—with HD patients also selectively showing lower initiation and poorer goal-directed planning than HCs. Higher apathetic features were detected in PD and HD as compared to ALS. Education was a protective factor against apathy in ALS. Anxiety was a risk factor for global apathy in ALS, HD, and to a lesser extent, in PD, whereas, protective only toward affective disintegration in PD and ALS. Cognitive inefficiency was a risk factor toward apathy in both PD and ALS. Depression was a risk factor for executive-related apathy in PD.

**Discussion:**

This study provides unprecedented insights into the heterogeneous semiology and determinants of apathy across ALS, PD, and HD *via* the DAS, in turn informing clinical practice and research.

## Background

Apathy is a behavioral alteration common to and highly prevalent in several neurodegenerative disorders (Dujardin, 2007; Starkstein et al., 2020) as a consequence of altered cortical and/or subcortical, frontal-parietal circuitries (Kos et al., 2016; Raimo et al., 2019). Its assessment is clinically crucial, as being a symptom that entails detrimental impacts on patients’ prognosis (van Reekum et al., 2005) and for whom promising both pharmacological (Bogdan et al., 2020) and non-pharmacological treatments (Manera et al., 2020) are available and/or in development. According to Robert et al. (2009) clinical *criteria*, such a disorder of motivation is diagnosed if (1) being persistent (i.e., ≥4 weeks), (2) entails at last two features among reduced cognitive/behavioral initiation or affective disintegration, (3) impairs individuals’ functional outcome, and (4) cannot be attributed to other causes (e.g., motor disability or depression).

However, detecting apathy in such populations represents a diagnostic challenge (Dujardin, 2007; Starkstein and Leentjens, 2008; Lanctôt et al., 2017), especially in respect to pyramidal (amyotrophic lateral sclerosis, ALS) and either hypokinetic (Parkinson’s disease, PD) or hyperkinetic (Huntington’s disease, HD) extrapyramidal disorders—due to the confounding effect of both motor disabilities and further neuropsychiatric symptoms (den Brok et al., 2015; Kutlubaev et al., 2022; Matmati et al., 2022). Although several nosographic systems and psychometric approaches have been proposed for detecting apathy, the multi-dimensional framework arguably represents the current gold-standard in clinical practice and research as addressed to neurodegenerative motor disorders (Cummings et al., 2015; Radakovic and Abrahams, 2018). Indeed, such a system, operationalized *via* the Dimensional Apathy Scale (DAS) (Radakovic and Abrahams, 2014), not only selectively captures the core features of apathy (Robert et al., 2009)—i.e., lack of cognitive and/or behavioral initiation, altered goal-directed planning and affective disintegration (i.e., emotional flattening/indifference)—, but also accommodates for motor disabilities, by not relying on observed variables associated with movement. The DAS has been indeed proved clinically feasible and useful in several brain disorders of diverse etiologies, besides ALS (Radakovic et al., 2016b,2017b; Santangelo et al., 2017c), PD (Santangelo et al., 2017a; Radakovic et al., 2018), and HD (Atkins et al., 2021)—e.g., Alzheimer’s disease (Radakovic et al., 2017a), multiple sclerosis (Raimo et al., 2020), frontotemporal degeneration (Radakovic et al., 2021), schizophrenia (M’Barek et al., 2021), and stroke (Myhre et al., 2022).

Despite much effort having been devoted to the study of apathy in ALS (Kutlubaev et al., 2022), PD (den Brok et al., 2015), and HD (Matmati et al., 2022), its semiology and determinants still remain to an extent elusive in these populations—likely due to the abovementioned confounders and subsequent measurement issues—, and no study to date has comparatively assessed it across these three conditions—in spite of the crucial diagnostic, and in turn interventional, entailments that both these investigations would convey. Indeed, apathy is believed to be moderately prevalent across all these disorders—ALS: ≈25–30% (Kutlubaev et al., 2022); PD: ≈40% (den Brok et al., 2015); HD: ≈30–50% (Matmati et al., 2022).

Given the above premises, this study thus aimed at (1) profiling apathy in ALS, PD, and HD patients within the multi-dimensional framework (i.e., through the DAS) (Kutlubaev et al., 2022) and (2) exploring it’s both motor and non-motor determinants separately for these three conditions.

## Methods

### Participants

Consecutive, clinically diagnosed ALS (*N* = 99) (Brooks et al., 2000), PD (*N* = 73) (Postuma et al., 2015), and HD (*N* = 25) patients (Reilmann et al., 2014) referred to IRCCS, Istituto Auxologico Italiano, Milan, Italy and LIRH Foundation, Rome, Italy between 2017 and 2022 were recruited, along with *N* = 101 healthy controls (HCs) (Table 1). Exclusion criteria were: (1) (other) neurological or psychiatric disorders; (2) severe general-medical conditions; (3) uncorrected hearing and/or vision deficits. This study was approved by the Ethics Committee of IRCCS Istituto Auxologico Italiano (I.D.: 2013_06_25) and by the Institutional Review Board of LIRH Foundation (I.D.: 1.010721); participants provided their informed consent and data were treated according to current regulations.

**TABLE 1:** Participants’ demographic and clinical measures.

	ALS	PD	HD	HCs	*P*
***N***	99	73	25	101	–
**Sex (M/F)**	51/48	51/22	16/9	39/62	HCs: F > M; PD: M > F^a^
**Age (years)**	63.99 ± 10.37(28–84)	68.59 ± 9.02 (47–90)	55.88 ± 16.07 (27–78)	63.39 ± 11.10 (24–86)	HD < HCs & ALS < PD^b^
**Education (years)**	12.52 ± 4.51 (5–19)	13.73 ± 3.68 (5–18)	11.76 ± 3.18 (8–17)	13.34 ± 3.80 (5–22)	n.s.^b^
**Disease duration (months)**	17.25 ± 17.87 (2–108)	82.63 ± 64.85 (6–288)	111.36 ± 164.41 (12–852)	–	PD & HD > ALS^c^
**ALSFRS-R**	39.50 ± 6.08 (22–48)	–	–	–	–
**UPDRS-II**	–	6.89 ± 5.44 (0–28)	–	–	–
**UPDRS-III**	–	13.05 ± 9.20 (0–39)	–	–	–
**UHDRS-I**	–	–	32.75 ± 13.53 (12–73)	–	–
**UHDRS-IV**	–	–	18.84 ± 4.50 (8–25)	–	–
**UHDRS-V**	–	–	80.80 ± 14.48 (50–100)	–	–
**UHDRS-VI**	–	–	9.24 ± 3.00 (3–13)	–	–
**HTT triplets (*N*)**	–	–	43.61 ± 4.51 (39–59)	–	–
**Genetics (*N*)**					–
C9orf72	3	–	–	–	–
TARDBP	3	–	–	–	–
SOD1	1	–	–	–	–
**King’s**					
Stage 0	4.9%	–	–	–	–
Stage 1	39%	–	–	–	–
Stage 2	25.6%	–	–	–	–
Stage 3	26.8%	–	–	–	–
Stage 4	3.7%	–	–	–	–
**Modified Hoehn-Yahr**					
Stage 1	–	15.3%	–	–	–
Stage 1.5	–	18.1%	–	–	–
Stage 2	–	34.7%	–	–	–
Stage 2.5	–	20.8%	–	–	–
Stage 3	–	11.1%			
**Shoulson-Fahn**					
Stage 1	–	–	36%	–	–
Stage 2	–	–	44%	–	–
Stage 3	–	–	20%	–	–
**ECAS**	100.73 ± 16.98 (43–127)	103.38 ± 14.03 (58–124)	81.20 ± 23.43 (34–116)	–	ALS & PD > HD^c^
**STAI-Y1**	53.74 ± 11.06 (33–87)	46.01 ± 10.33 (20–78)	51.60 ± 9.37 (37–76)	–	ALS > PD^b^
**STAI-Y2**	49.54 ± 9.32 (33–71)	48.26 ± 9.43 (34–74)	49.52 ± 8.22 (35–63)	–	n.s.^b^
**BDI**	13.33 ± 8.62 (0–41)	9.21 ± 3.38 (0–38)	10.24 ± 7.17 (0–25)	–	ALS > PD^b^
**DAS**					
Total	21.52 ± 7.51 (3–40)	24.19 ± 7.15 (11–41)	29.20 ± 9.09 (11–48)	20.69 ± 7.42 (1–55)	PD & HD > HCs^b^
Initiation	8.40 ± 4.51 (0–20)	8.73 ± 4.16 (1–23)	10.32 ± 5.62 (2–23)	7.36 ± 3.38 (0–17)	HD > HCs^b^
Executive	6.63 ± 4.21 (0–18)	7.44 ± 4.29 (0–19)	12.00 ± 5.01 (6–24)	6.43 ± 4.11 (0–26)	HD > HCs^b^
Emotional	6.48 ± 3.50 (0–15)	8.03 ± 3.21 (0–15)	6.88 ± 3.13 (2–14)	6.86 ± 3.48 (0–21)	n.s.

ALS, amyotrophic laterals sclerosis; ALSFRS-R, amyotrophic lateral sclerosis functional rating scale-revised; BDI, Beck depression inventory; DAS, dimensional apathy scale; ECAS, Edinburgh cognitive and behavioral ALS screen; F, female; HCs, healthy controls; HD, Huntington’s disease; HTT, huntingtin; M, male; PD, Parkinson’s disease; STAI-Y1, state and trait anxiety inventory–form Y–state anxiety; STAI-Y2, state and trait anxiety inventory–form Y–trait anxiety; UHDRS, unified Huntington’s disease rating scale; UPDRS, unified Parkinson’s disease rating scale. ^a^Adjusted standardized residuals (χ^2^-statistics). ^b^Bonferroni-corrected *post-hoc* comparisons (*F*-statistics). ^c^Dwass-Steel-Critchlow-Flinger *post-hoc* comparisons (Kruskall-Wallis *H*-statistics).

### Materials

Both patients and HCs were administered the DAS (Santangelo et al., 2017b), a self-report questionnaire subdivided into three subscales tapping on Initiation (*range*: 0–27), Executive (*range*: 0–27) and Emotional dimensions (*range*: 0–18) of apathy (total score *range*: 0–72; higher scores corresponding to higher levels of apathetic features). Furthermore, patients underwent an *ad hoc* cognitive, behavioral and functional assessment. Global cognition was assessed *via* the cognitive section of the Edinburgh Cognitive and Behavioral ALS Screen (ECAS) (Poletti et al., 2016), a ALS-specific, performance-based screener—which has nevertheless shown sound diagnostics also in PD and HD patients (Carelli et al., 2021)—assessing attention/executive functioning, language, memory, and visuo-spatial abilities (*range* = 0–136). Anxiety was assessed *via* the State-Trait Anxiety Inventory-Y (STAI-Y1 and –Y2 for state- and trait-anxiety, respectively), a self-report, 40-item questionnaire (20 items assessing state- and trait-anxiety, respectively) (Spielberger et al., 1971) and depression *via* the Beck Depression Inventory (BDI), a self-report, 21-item questionnaire assessing cognitive and somatic dimension of depression (Beck et al., 1961). Function was assessed through disease-specific scales—ALS: ALS Functional Rating Scale-Revised (ALSFRS-R), assessing motor-functional outcomes in daily living (Cedarbaum et al., 1999); PD: Unified Parkinson’s Disease Rating Scale (UPDRS)-II (assessing motor function) and –III (assessing functional independence) (Fahn et al., 1987); HD: Unified Huntington’s Disease Rating Scale (UHDRS)-I (assessing motor function) as well as –IV, –V, and –VI (assessing functional independence) (Huntington Study Group, 1996). Staging was derived through *ad hoc* systems—ALS: King’s staging (Roche et al., 2012); PD: modified Hoehn-Yahr staging (Fahn and Elton, 1987); HD: Shoulson-Fahn staging (Shoulson and Fahn, 1979)—with higher scores indexing more advanced disease stages.

### Statistics

Normality and heteroscedasticity assumptions—as assessed by means of skewness and kurtosis values (judged as normal if < |1| and |3|, respectively), histograms and quantile-quantile plots and Kolmogorov-Smirnov statistics (Kim, 2013)—were met for all DAS raw scores. Thereupon, linear model analyses were performed to test associations/predictions of interest.

Comparisons between HCs and each clinical group on DAS subscales and its total score were performed *via* a multivariate and a univariate analysis of variance, respectively—both followed by planned comparisons (i.e., HCs *vs.* each clinical group). Since the four groups were unbalanced for age and sex, these variables were covaried within these models.

The same analyses were performed to selectively compare the three clinical cohorts, by covarying for additional unbalanced variables (besides age and sex; i.e., disease duration as well as ECAS, STAI-Y1, and BDI scores) and followed by Bonferroni-corrected *post-hoc* comparisons.

To explore the determinants of DAS scores in each clinical group, stepwise, multiple linear regressions were run separately for each subscale and the total score by entering, as predictors, age, education, sex, disease duration as well cognitive/behavioral (ECAS; STAI-Y1; STAI-Y2; BDI) and functional measures (ALSFRS-R, UPDRS-II/-III, UHDRS-I/-IV/-V/-VI). In order to control for type-I error inflation rates, the α-level was Bonferroni-corrected as follows: α_*adjusted*_ = α/(k*N_*i*_), where *k* is a constant equal to the number of groups (i.e., *N* = 3: ALS, PD and HD) and *N*_*i*_ is the number of significant predictors (*p* < 0.05) yielded by the final regression step within a given *i*th model.

Analyses were run with R 4.1^1^ and jamovi 2.3 (the jamovi project, 2022).

## Results

Table 1 summarizes participants’ background and clinical variables. Overall, mild-to-moderate clinical stages were similarly represented in all clinical groups, with a relative small proportion of patients reaching the final ones and the median stage being 2 for all cohorts.

Planned comparisons on the DAS-Total revealed, in spite of a marginally significant, overall effect of group [*F*_(1,292)_ = 3.8; *p* = 0.052], that HCs were comparable to the ALS cohort but scored lower than both PD [*F*_(1,292)_ = 4.29; *p* = 0.039] and HD patients [*F*_(1,292)_ = 26.43; *p* < 0.001]. As to DAS subscales, an *omnibus* group effect was detected [Wilk’s λ = 0.85; *F*_(9,705.93)_ = 5.47; *p* < 0.001], being accounted for, at planned comparisons, by HCs reporting lower DAS-Executive [*F*_(1,292)_ = 36.64; *p* < 0.001] and DAS-Initiation scores [*F*_(1,292)_ = 11.75; *p* < 0.001] than HD patients—with other comparisons (i.e., ALS *vs.* HCs and PD *vs.* HCs) yielding no significance on any of the DAS subscales.

As to comparisons between clinical cohorts, a group effect was detected for the DAS-Total [*F*_(1,186)_ = 9.02; *p* < 0.001], which proved to be carried *a posteriori* by the significant differences (*p* ≤ 0.009) between ALS (*M* = 21.02; *SE* = 0.8) and both PD (*M* = 24.96; *SE* = 0.94) and HD patients (*M* = 28.48; *SE* = 1.69). As to DAS subscales, an overall group effect yielded [Wilk’s λ = 0.86; *F*_(6,368)_ = 4.67; *p* < 0.001], whose *post-hoc* decomposition revealed that ALS patients differed from both PD (*p* = 0.001) and HD ones (*p* < 0.001) on the DAS-Executive (ALS: *M* = 6.13; *SE* = 0.42; PD: *M* = 8.62; *SE* = 0.49; HD: *M* = 10.63; *SE* = 0.89), as well as, from the HD cohort (*p* = 0.031), on the DAS-Initiation (ALS: *M* = 8.03; *SE* = 0.49; HD: *M* = 11.15; *SE* = 1.04)—with no other comparisons, on either the DAS-Initiation or the DAS-Emotional, being significant.

Table 2 reports the results of the stepwise regression procedures. In ALS patients, education negatively predicted DAS-Total and DAS-Initiation scores, the ECAS negatively predicted the DAS-Executive, the STAI-Y1 negatively predicted the DAS Emotional and the STAI-Y2 positively predicted DAS-Total, DAS-Initiation and DAS-Executive scores. As to the PD cohort, the ECAS negatively predicted DAS-Total and DAS-Emotional scores, the STAI-Y2 negatively predicted the DAS-Emotional, the STAI-Y1 positively predicted the DAS-Initiation and the BDI positively predicted the DAS-Executive. Only the STAI-Y2 positively predicted DAS-Total and DAS-Initiation scores in HD patients—with no other significant predictors yielding as to DAS-Executive and DAS-Emotional ones.

**TABLE 2:** Determinants of dimensional apathy scale (DAS) scores in amyotrophic lateral sclerosis (ALS), Parkinson’s disease (PD), and Huntington’s disease (HD) patients.

Group	Predictors	DAS-total		DAS-initiation		DAS-executive		DAS-emotional	
					
		β	*P*	β	*P*	β	*P*	β	*P*
ALS	Education	**-0.48**	0.006^b^	**-0.53**	<0.001^d^	n.e.	–	n.e.	–
	ECAS	n.e.	–	0.25	0.009	**-0.37**	<0.001^c^	n.e.	–
	STAI-Y1	n.e.	–	n.e.	–	n.e.	–	**-0.3**	0.006^a^
	STAI-Y2	**0.36**	<0.001^b^	**0.35**	<0.001^d^	**0.39**	<0.001^c^	n.e.	–
	BDI	n.e.	–	n.e.	–	0.24	0.013	n.e.	–
	ALSFRS-R	n.e.	–	–0.25	0.009	n.e.	–	n.e.	–
PD	Education	n.e.	–	n.e.	–	n.e.	–	n.e.	–
	ECAS	**-0.4**	<0.001^b^	n.e.	–	–0.27	0.010	**-0.31**	0.005^b^
	STAI-Y1	n.e.	–	**0.28**	0.016^a^	n.e.	–	n.e.	–
	STAI-Y2	n.e.	–	n.e.	–	n.e.	–	**-0.33**	0.004^b^
	BDI	0.24	0.023	n.e.	–	**0.42**	<0.001^b^	n.e.	–
HD	Education	n.e.	–	n.e.	–	n.e.	–	n.e.	–
	ECAS	n.e.	–	n.e.	–	n.e.	–	n.e.	–
	STAI-Y1	n.e.	–	n.e.	–	n.e.	–	n.e.	–
	STAI-Y2	**0.56**	0.005^a^	**0.65**	0.001^a^	n.e.	–	n.e.	–
	BDI	n.e.	–	n.e.	–	n.e.	–	n.e.	–
	UHDRS-VI	n.e.	–	n.e.	–	–0.48	0.017	n.e.	–

ALS, amyotrophic laterals sclerosis; BDI, Beck depression inventory; DAS, dimensional apathy scale; ECAS, Edinburgh cognitive and behavioral ALS screen; HD, Huntington’s disease; n.e., predictor not entered into the final step of the stepwise regression procedure; PD, Parkinson’s disease; STAI-Y1, state and trait anxiety inventory–form Y–state anxiety; STAI-Y2, state and trait anxiety inventory–form Y–trait anxiety. Significant regression coefficients at respective, adjusted α thresholds are in bold. ^a^Significant at α_adjusted_ = 0.017. ^b^Significant at α_adjusted_ = 0.008. ^c^Significant at α_adjusted_ = 0.006. ^d^Significant at α_adjusted_ = 0.004. For each group, only those predictors entered into the final step of at least one of the model are shown.

## Discussion

The present study provides further insights into the semiology and determinants of apathy in ALS, PD and HD, also comparing it across these three populations, by means of the DAS—a psychometric tool selectively assessing the core dimensions of apathy (i.e., lack of cognitive/behavioral initiation, altered goal-directed planning and affective disintegration) by accommodating for motor disabilities (Radakovic and Abrahams, 2014).

Overall, PD and HD patients proved to show more prominent apathetic features when compared to HCs, although no differences have been detected between the latter and ALS patients.

This last finding is in line with previous studies by Radakovic et al. (2016b,2017b) as to the DAS-Total being unable to discriminate between ALS patients and HCs, whereas, at the same time, in contrast with them as to the fact that certain DAS subscales (i.e., the DAS-Initiation and the DAS-Emotional) have been shown to be able to do so. Nevertheless, it behoves noting that the present study included a greater number of ALS patients and HCs than Radakovic et al. (2016b,2017b) ones—this supporting that the results yielding from the former have an higher degree of generalizability when compared to those yielding from the latter. At the same time, the inability of the DAS to differentiate ALS patients from HCs has been herewith reported at a group level: thereupon, such a finding should not lead to incautiously infer that the prevalence of clinically meaningful apathetic features in ALS patients, as revealed by the DAS, is comparable to that detectable in the general population—since this could be only determined by comparing the prevalence of abnormal DAS scores in ALS patients and HCs as yielded by their respective, group-specific normative cutoffs (Santangelo et al., 2017b,c). However, such an analysis was not intended to be performed within the present study, and should be thus addressed in future ones.

As to the comparisons between HCs and PD and HD patients, the finding of overall apathy levels being higher in the latter groups as compared to the former is in line with the concerning literature, which acknowledges apathy as typical of these patients’ behavioral profile (den Brok et al., 2015; Matmati et al., 2022).

However, when specifically addressing DAS subscales, PD patients did not differ from HCs on any of them—this suggesting that, in this population, there might not be specific dimensions contributing to the emergence of apathy (which would, by contrast, feature itself as a generalized disorder of motivation). Such findings are to an extent in line with previous studies comparing PD patients and HCs on DAS scores (Santangelo et al., 2017a; Radakovic et al., 2018), reporting that each DAS subscale has the potential to discriminate PD patients from HCs—this suggesting that no specific apathy dimensions are accountable for such case-control differences.

As to HD patients, the present study agrees with a recent report by Atkins et al. (2021)—which is the only one that has previously compared HD patients to HCs on DAS scores—as to the fact that a lack of cognitive/behavioral initiation and an altered goal-directed planning contribute to the emergence of apathy in this population.

The present findings are unprecedented as to the comparison between ALS, PD and HD patients on DAS score, showing that ALS patients present with (1) lower global and executive-related apathetic features than PD and HD ones and (2) a higher cognitive/behavioral initiation than HD patients. Thereupon, it can be hypothesized that an alteration of goal-directed planning and a lack of cognitive/behavioral initiation predominantly feature the clinical presentation of apathy in extrapyramidal, hypokinetic/hyperkinetic disorders—at least when compared to ALS. It is beyond the scopes of this study to provide insights into the neural correlates of such a finding. However, it can be likewise speculated that the higher level of such apathetic features in HD and PD as compared to ALS might be accounted for by the direct involvement of nigro-striatal structures, which represents the neural hallmark of the former disorders (Santangelo et al., 2013; De Paepe et al., 2019)—at variance with ALS, where apathy appears to indirectly yield from a disruption of a widespread network of frontal-parietal, cortical areas (Femiano et al., 2018; Caga et al., 2021; Canosa et al., 2021b). Further research is needed to test such a hypothesis.

Finally, intricate and substantially novel results have been herewith reported as to the determinants of apathy across ALS, PD, and HD.

With regard to ALS patients, higher education being predictive of lower DAS-Total and DAS-Initiation scores is a previously unreported finding. However, it should be noted that, in the general population, higher education was found to be the sole, demographic predictor of lower DAS-Total scores (Santangelo et al., 2017b), as well as that cognitive reserve (which is strictly related to and inclusive of education) can protect against apathy (Altieri et al., 2020). Moreover, such an interplay between education and apathy has been reported also in PD patients (Pedersen et al., 2009; Cubo et al., 2012; Gorzkowska et al., 2021), and cognitive reserve has been found to be protective against apathy in patients with HIV—and thus at risk for encephalopathic sequelae (Shapiro et al., 2014). Finally, education has been proposed to influence behavioral phenotypes of patients with behavioral variant-frontotemporal dementia by means of articulate cortical reorganization processes (Premi et al., 2013). By putting together these reports with recent evidence suggestive of the role played by education/cognitive reserve toward cognitive phenotypes in ALS patients (Canosa et al., 2021a; Consonni et al., 2021; Costello et al., 2021; Temp et al., 2021), it can be hypothesized that, in this population, education (either by itself or as regarded as a proxy of cognitive reserve) is protective against the emergence of apathetic features.

The finding of higher cognitive efficiency (ECAS) being linked to lower DAS-Executive in ALS patients and to lower DAS-Total and DAS-Emotional scores in PD ones is in line with meta-analytic evidence identifying that cognitive impairment can be associated with apathy in ALS (Kutlubaev et al., 2022) and PD (den Brok et al., 2015). However, within this study, such a link has been further unraveled—indeed, in ALS patients, cognitive performance appeared to be specifically related to goal-directed planning, whereas, in PD ones, to overall apathetic features and affective disintegration.

Notably, only in PD patients, higher levels of depression (BDI) proved to be predictive of higher DAS-Executive scores. Such a finding is only partially consistent with previous reports addressing the link between DAS scores and depression measures in this population (Santangelo et al., 2017a; Radakovic et al., 2018)—which found also DAS-Total scores and other DAS subscales to be associated with depressive symptoms. However, it should be noted that such studies (Santangelo et al., 2017a; Radakovic et al., 2018) reported only simple correlational analyses and did not include multiple predictive models—as is the case for the present work. Therefore, given that, on one hand, the association between depression and apathy in PD patients is frequent (Isella et al., 2002; Oguru et al., 2010; Santangelo et al., 2013; Macías-García et al., 2022), whereas that, on the other, the two clusters can be dissociable in this population (Isella et al., 2002; Kirsch-Darrow et al., 2006, 2011; Skidmore et al., 2013), it would not be incautious to hypothesize that, in PD patients, the contribution of depression to apathy is selective to the goal-oriented planning dimension of the latter.

Finally, the present study highlights for the first time the multi-faceted role of anxiety as a determinant of apathy in ALS, PD, and HD.

Indeed, higher trait-anxiety levels (STAI-Y2) proved to be predictive of higher DAS-Total/-Executive/-Initiation scores in ALS patients, as well as the only predictor of higher DAS-Total/-Initiation scores in the HD cohort. Moreover, only in PD patients, higher state-anxiety levels (STAI-Y1) predicted higher DAS-Initiation scores. Finally, lower DAS-Emotional scores were predicted by higher trait-anxiety levels in PD patients, whereas by higher state-anxiety levels in ALS ones.

The positive association between the STAI-Y2 and DAS scores in ALS patients is consistent with Siciliano et al. (2019) correlational findings, despite being unprecedented when compared to them as yielding, within the present study, from multiple predictive models. By contrast, similar findings as referred to HD patients have never been previously reported—and, conversely, anxiety has been postulated to be mostly unrelated to apathy in this population (Dale and van Duijn, 2015). Given the novelty of these results, further research in undoubtedly needed in order to unravel the actual role of trait-anxiety in determining the emergence of apathy in ALS and HD patients. However, recent records addressing another neurodegenerative disorder, i.e., Alzheimer’s disease, suggest that anxiety and apathy might share common neurochemical grounds (Johansson et al., 2020), as well as that trait-anxiety may concur to the pathophysiology of apathy *via* neuroendocrine and neurochemical alterations (Li et al., 2021). Moreover, higher trait-anxiety levels have been related to more prominent apathetic features in PD patients, with this association being underpinned by a specific serotonergic dysfunction located in the striatum and nearby limbic structures (Maillet et al., 2016). Therefore, it can be speculated that similar, multi-faceted pathophysiological mechanisms underlying trait-anxiety might account for brain abnormalities in turn contributing to the emergence of apathetic features also in ALS and HD.

Also the predictive capability of state-anxiety toward executive-related apathetic features herewith reported in PD patients is unprecedented. In support to such a finding, an association between apathy levels and overall measures of anxiety has been previously reported (Santangelo et al., 2016; Radakovic et al., 2018). By expanding this evidence, it can be thus hypothesized that state-anxiety represents a risk factor specifically for a poor goal-directed planning in PD.

Finally, the counterintuitive protective role toward affective disintegration of trait-anxiety in PD patients, as well as that of state-anxiety in ALS ones, might be explained by the fact that more anxious patients are more reactive and less indifferent to emotional stimuli within their environment. Since no direct evidence is available that supports this last hypothesis, it can be also postulated, based on a recent meta-analytic work addressing mild-to-moderate Alzheimer’s disease patients (Azocar et al., 2021) that, across neurodegenerative disorders, the inverse association between anxiety and affective disintegration may be mediated by anosognosic features. Indeed, within the abovementioned meta-analysis (Azocar et al., 2021), it is proposed that, in early Alzheimer’s disease, impaired awareness is linked to lower anxiety, but higher apathy, levels. Future studies are therefore needed in order to explore the potential role of disease awareness in accounting for the association between anxiety and apathy in PD and ALS.

As to the fact that neither function nor disease duration was related to apathetic features in any of the three cohorts, this supports the notion of the DAS being actually a motor-free measure of apathy (Radakovic and Abrahams, 2014; Santangelo et al., 2017b).

The present study is of course not free of limitations. First, the sample sizes were unequal across the four groups, with HD patients being under-represented—this to an extent limiting the external validity of findings that address them. Furthermore, the present work is not exhaustive of all the possible contributors to apathy—e.g., measures of overall behavioral dysfunction and of awareness, as well as second-level, domain-/function-specific measures of cognition—and, due to its cross-sectional nature, it does not allow to draw inferences on the capability of the addressed determinants to predict the course of apathetic features over time. Additionally, despite cognition and behavior have been accounted for *via* continuous measure, future investigations should focus on exploring the semiology and determinants of apathy in ALS by stratifying patients according to the current, cognitive/behavioral nosographic system (Strong et al., 2017). Finally, this study was solely based on psychometric measures and did not address biomarkers, this not allowing to conclude on the neural underpinnings of the findings herewith reported.

## Conclusion

This study provides further and novel insights into the clinical presentation and predictors of apathy across ALS, PD, and HD by means of specific apathy measures that control for motor disabilities (DAS), thus increasing the knowledge on its semiology and determinants in these populations and, in turn, informing clinical practice and research as addressed to them.

Parkinson’s disease and HD, but not ALS, patients were more apathetic than HCs. Moreover, when compared to HCs, HD patients selectively showed lower cognitive/behavioral initiation and poorer goal-directed planning. ALS patients showed lower apathy levels than PD and HD ones, especially in respect to the executive- and initiation-related apathetic dimensions.

Education was a protective factor against apathy in the ALS cohort. Higher trait-anxiety levels overall predicted higher apathetic features in ALS and HD patients; by contrast, higher trait- and state-anxiety levels were protective against affective disintegration in PD and ALS patients, respectively. In PD patients, global apathy was predicted by lower cognitive levels. Cognitive inefficiency was selectively predictive of poor goal-directed planning in ALS patients. Finally, depression was a risk factor for executive-related apathy only within the PD cohort.

In conclusion, across ALS, PD, and HD, apathetic profiles are not overlapping and the determinants of apathy are highly heterogeneous—this prompting further, comparative research on these topics.

## Data availability statement

The raw data supporting the conclusions of this article will be made available by the authors, without undue reservation.

## Ethics statement

The studies involving human participants were reviewed and approved by the Ethics Committee of IRCCS Istituto Auxologico Italiano (I.D.: 2013_06_25) and by the Institutional Review Board of LIRH Foundation (I.D.: 1.010721). The patients/participants provided their written informed consent to participate in this study.

## Author contributions

BP, SiM, FV, VS, NT, FSq, and AC: conceptualization, resources, drafting, and revision. EA: analyses, drafting, and revision. FSo, ST, LC, SaM, ES, and MC: data collection and revision. RF, AP, and AM: revision. All authors contributed to the article and approved the submitted version.

## Footnotes

1
https://cran.r-project.org/
